# *In vivo* Bioluminescence Imaging of Ca^2+^ Signalling in the Brain of *Drosophila*

**DOI:** 10.1371/journal.pone.0000275

**Published:** 2007-03-07

**Authors:** Jean-René Martin, Kelly L. Rogers, Carine Chagneau, Philippe Brûlet

**Affiliations:** 1 Equipe: Bases Neurales des Comportements chez la Drosophile, Laboratoire de Neurobiologie Cellulaire et Moléculaire (NBCM), Centre National de la Recherche Scientifique (CNRS), Unité UPR-9040, Gif-sur-Yvette, France; 2 Unité d'Embryologie Moléculaire, Centre National de la Recherche Scientifique (CNRS), URA 2578, Institut Pasteur, Paris, France; 3 Neurobiologie de l'Apprentissage, de la Mémoire et de la Communication (NAMC), Centre National de la Recherche Scientifique (CNRS), UMR-8620, Université Paris-Sud, Orsay, France; Katholieke Universiteit Leuven, Belgium

## Abstract

Many different cells' signalling pathways are universally regulated by Ca^2+^ concentration [Ca^2+^] rises that have highly variable amplitudes and kinetic properties. Optical imaging can provide the means to characterise both the temporal and spatial aspects of Ca^2+^ signals involved in neurophysiological functions. New methods for *in vivo* imaging of Ca^2+^ signalling in the brain of *Drosophila* are required for probing the different dynamic aspects of this system. In studies here, whole brain Ca^2+^ imaging was performed on transgenic flies with targeted expression of the bioluminescent Ca^2+^ reporter GFP-aequorin (GA) in different neural structures. A photon counting based technique was used to undertake continuous recordings of cytosolic [Ca^2+^] over hours. Time integrals for reconstructing images and analysis of the data were selected offline according to the signal intensity. This approach allowed a unique Ca^2+^ response associated with cholinergic transmission to be identified by whole brain imaging of specific neural structures. Notably, [Ca^2+^] transients in the Mushroom Bodies (MBs) following nicotine stimulation were accompanied by a delayed secondary [Ca^2+^] rise (up to 15 min. later) in the MB lobes. The delayed response was sensitive to thapsigargin, suggesting a role for intra-cellular Ca^2+^ stores. Moreover, it was reduced in *dunce* mutant flies, which are impaired in learning and memory. Bioluminescence imaging is therefore useful for studying Ca^2+^ signalling pathways and for functional mapping of neurophysiological processes in the fly brain.

## Introduction

Homeostatic mechanisms ensure that Ca^2+^ ions in the intracellular millieu always exist in a dynamic state of motion and any sudden rise in [Ca^2+^] is tightly regulated in both space and time. Increases in intracellular [Ca^2+^] can reach very high concentrations in cellular microdomains in which Ca^2+^-dependent processes can be both rapidly and selectively activated. These kind of fast Ca^2+^ transients regulate cellular events at the synaptic terminal, such as exocytosis. However, Ca^2+^ oscillations or slow Ca^2+^ waves that propagate across cells or tissues, can also drive functions such as gene expression. The widely different dynamic properties of Ca^2+^ signalling forms the basis of how Ca^2+^ ions are able to be a universal regulator of many signal transduction pathways [1], [2].

The spatiotemporal properties of Ca^2+^ signals can be characterised by optical imaging. However, the choice of Ca^2+^ sensitive fluorescent or bioluminescent probe will introduce a bias in optical imaging studies, because the signal output will be dependent on a number of important factors including; the Ca^2+^ binding affinity, the intrinsic properties of the Ca^2+^ induced light reaction, whether or not and where the probe is genetically targeted, the sensitivity of the technique, the size of the field of view and the pre-selection of how data will be acquired (i.e. how the spatial and temporal parameters of the study are defined in the acquisition protocol).

*In vivo* Ca^2+^-imaging in the brain of *Drosophila* with genetically encoded Ca^2+^ sensitive fluorescent reporters, such as cameleon [3], [4], camgaroo [5], and G-CaMP [6], [7], have given new information about neural mechanisms underlying olfactory learning and memory. Although these approaches provide excellent spatio-temporal resolution of Ca^2+^ signals, excitation light is required and this introduces some limitations relating to photo-toxicity, photobleaching and autofluorescence. Furthermore, when fluorescent probes are used, it is necessary to pre-define the temporal parameters of each study and studies are often designed with the expectation that a Ca^2+^ response with certain temporal parameters will follow a given stimulus (e.g. application of NMDA or stimulation with different odors). Hence, Ca^2+^ signals that occur long after a given stimulus in living biological systems are not well described [8].

Bioluminescence imaging of Ca^2+^ signals is not associated with phototoxicity and provides an excellent signal-to-noise ratio. GFP-aequorin (GA) is a Ca^2+^ sensitive bioluminescent photoprotein with improved light emission properties compared to aequorin alone [9], [10]. The bioluminescence reaction of GA occurs within milliseconds after Ca^2+^ binding [11], and has fast enough kinetics to allow changes in [Ca^2+^] to be followed over a wide dynamic time range, from milliseconds to hours [10] (and unpublished data). GA is also insensitive to pH in the physiological range [12].

Here, we demonstrate the use of *in vivo* bioluminescence imaging of Ca^2+^ signaling in the brain of *Drosophila.* Whole brain imaging was undertaken on transgenic flies with targeted expression of the GA probe in specific neural structures, such as Mushrooms Bodies and ellipsoid-body (a substructure of the Central Complex). A photon counting based technique was applied to avoid the necessity to pre-select temporal parameters and to provide a wide dynamic range for detecting Ca^2+^ signals. In conclusion, bioluminescence imaging of Ca^2+^ signals permits the imaging of neuronal ensembles in deep regions of the brain and gives great flexibility to analyse the temporal parameters of Ca^2+^ signaling over long durations.

## Results

### Detection of Ca^2+^ dynamics in flies expressing GFP-aequorin in the MBs

**Figure 1:**
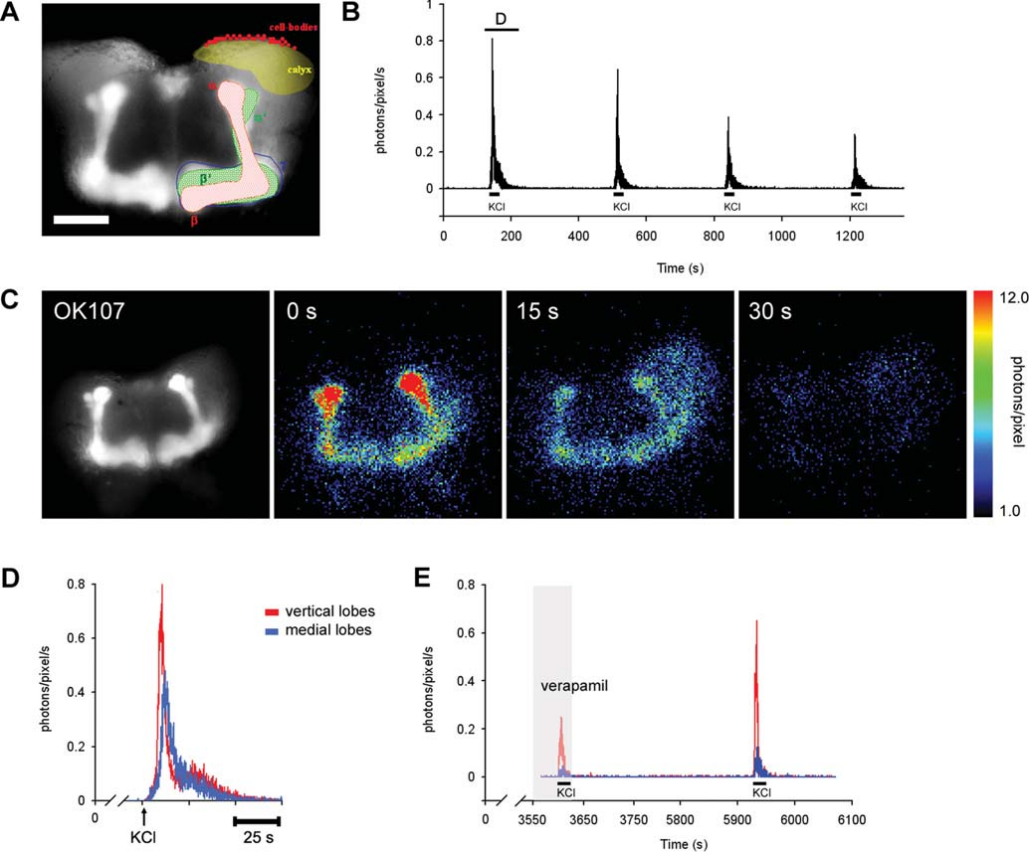
*In vivo* bioluminescence imaging of Ca^2+^-dynamics in the MBs. A) Fluorescence image superimposed with a schematic drawing of wild-type Canton-S MBs. GFP-aequorin (GA) expression in the MBs was driven by the P[GAL4]OK107 line. Seen are the vertical and medial lobes, which are localised in the anterior part of the brain. They are subdivided into five axonal lobes; α and β lobes (in red), α' and β' (in green), and the γ lobe (in blue) (note that the γ lobe does not have a vertical counterpart and the tip of the α' lobe terminate laterally to the α lobe). The calyx (in yellow shading) and the cell bodies (in magenta) are located dorsally and posteriorly in the brain. Scale bar = 50 µm. B) Light emission (photons/pixel/s) plotted as a function of time (s), following four successive applications of high [KCl]. C) A representative example of K^+^-depolarised Ca^2+^ influx in the MBs. The first frame shows an image of the whole brain and the localization of GFP fluorescence in the MBs. Following frames show consecutive bioluminescent images (15 s integration) after K^+^-depolarization (70 mM), starting at “0” when KCl was applied. D) Light emission from each lobe (see in C) was plotted as a function of time (photons/pixel/s). The red trace corresponds to the tip of the vertical lobes (α, α' lobes), the blue trace to the medial (β, β') lobes. E) The first peak shows the effect verapamil (10 µM) on the K^+^-depolarized Ca^2+^-response in the lobes. The second peak shows K^+^-depolarised Ca^2+^-uptake in the lobes after washout of the drug. Red trace, vertical lobes and blue trace is medial lobes.

GA transgenic flies were developed with the P[GAL4]/UAS system [13], in order to specifically target GA to neuronal subsets. The sensitivity of *in vivo* imaging with GA was first determined by targeting the Ca^2+^ reporter to the MBs using the P[GAL4] line OK107 [7] (Figure 1A, and for a more complete description of the fly brain anatomy, see ref. [14]). For comparison with fluorescence approaches, we began these studies by using similar protocols as those described previously [4], [5], and Ca^2+^-responses were recorded with whole brain bioluminescence imaging after a short bath application of KCl (70 mM) (Figure 1B-D, movie S1). At low magnification, K^+^-depolarised Ca^2+^-uptake in the MBs produced a robust signal above background that could be optically detected in all parts of the KCs (calyx, cell bodies and lobes). K^+^-depolarised Ca^2+^ responses in the MBs were also reduced by the L-type voltage-gated calcium channel (VGCC) blocker, verapamil (10 µM) (Figure 1E). During these studies, we also observed small amounts of spontaneous activity (referred here as non-induced activity) in all parts of the MBs, as well as on some occasions was also observed unilaterally (data not shown).

### Functional imaging of the ellipsoid-body

In *Drosophila*, the central-complex (CC) is composed of small cells located deep inside the middle of the brain (for a complete description of the CC's anatomy, see ref. [15]). Other than insight gathered from genetic techniques (e.g. studies on mutants) [16]–[18], there are no reports detailing electrophysiological or functional characteristics of this structure in *Drosophila* and there is a general lack of functional information for this part of the brain.

**Figure 2:**
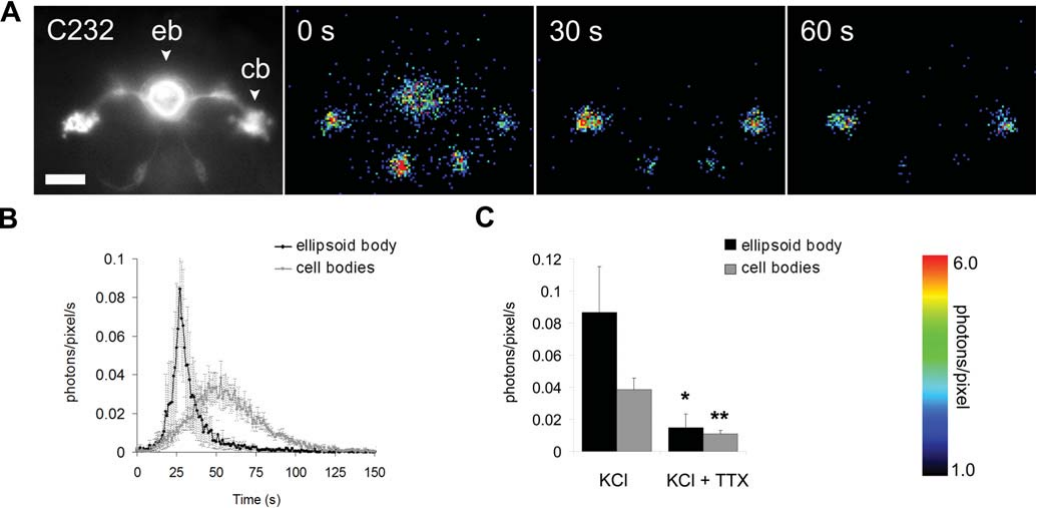
*In vivo* detection of Ca^2+^ activities in the ellipsoid-body (eb). A) A representative example showing GFP fluorescence of GA expression in the eb (P[GAL4] C232), a sub-structure of the CC. This frontal view shows the cell bodies (cb), which are localized frontally, medially and laterally to the eb structure. The ring-shaped arborisation of the ring-neurons is also visible in the center of the brain. The cell bodies are on a different plane and are therefore slightly out of focus. Outside the eb, few other neurons located ventrally to the eb, are also labeled. However, those neurons are not yet precisely characterized. (20× objective, scale bar = 50 µm). Corresponding frames show Ca^2+^-induced bioluminescence after K^+^-depolarisation (70 mM). Each image represents 30 s of accumulated light, starting at “0” when KCl was applied. B) Traces showing the average light emission±s.e.m in the eb and the cell bodies (photons/pixel/s, n = 3 flies). C) Histogram represents the calculated means+/−s.e.m. for the maximum photons/pixel/s determined for the eb (n = 3 flies) and cell bodies with and without TTX (n = 6, 3 flies). *P<0.05, **P<0.01.

GA expression was therefore targeted to a subset of ring-neurons in the ellipsoid-body (eb) (Figure 2A), using the P[GAL4]C232 line [17], [18]. Application of high K^+^ evoked elevations in [Ca^2+^]_i_ within the eb and cell bodies (Figure 2A–C, movie S2), which were readily detectable by the GA probe. The response was also significantly attenuated in the presence of tetrodotoxin (TTX), which is a blocker of voltage-gated Na^+^ channels. Ca^2+^ responses were therefore largely due to activation of neurons (pre-synaptic) that send projections to the eb (Figure 2C). This result also confirms that GA is sensitive enough to follow trans-synaptic activation and therefore could be use to functionally map neuronal circuitry. During prolonged whole brain recordings (for several hours), similarly to the MBs, spontaneous activity has also been observed in the eb according to where GA had been genetically targeted (movie S3).

### New insight on cholinergic induced Ca^2+^-signalling in the MBs

**Figure 3:**
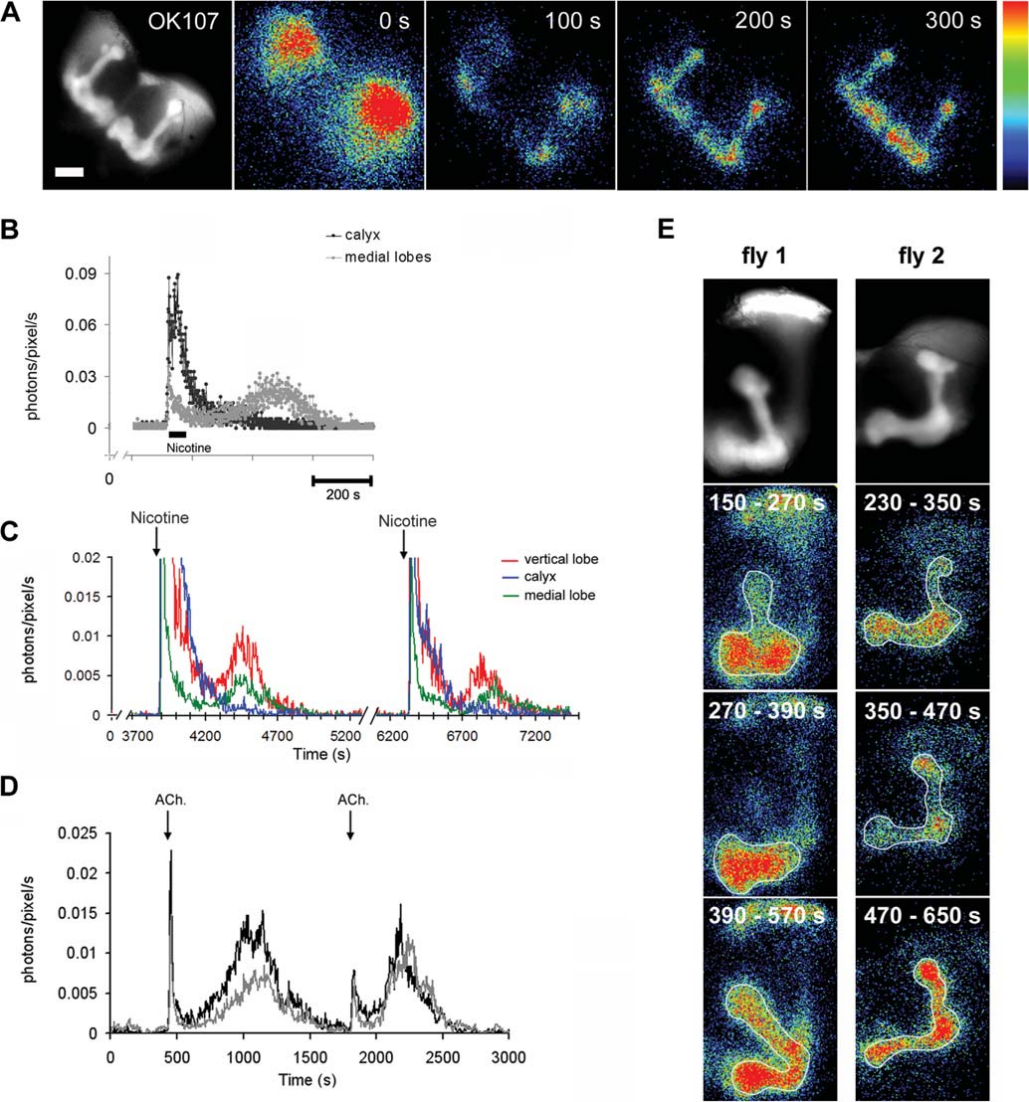
Nicotine induced [Ca^2+^] signalling in the MBs. A) Representative example of GFP fluorescence in the whole brain of *Drosophila* expressing GA in the MBs (scale bar = 50 µm). Corresponding frames show consecutive images of bioluminescence, after application of nicotine (100 µM). Note that the secondary rise in [Ca^2+^]_i_ has a slow rate of rise and decay in the vertical (α, α') and medial (β, β' and γ) lobes. Bioluminescence images each represent 100 s of accumulated light, starting at “0” when nicotine was applied. B) Traces showing the light emission versus time from the calyx-cell bodies and the medial lobes in (A). C) Traces showing that the secondary Ca^2+^-response can be reproduced after sequential application of nicotine (100 µM). D) Light emission in the medial lobes after two consecutive applications of acetylcholine (100 µM). E) Two representative examples (fly 1 & 2) of Ca^2+^-induced bioluminescence in the lobes after application of nicotine (100 µM) showing that the delayed secondary Ca^2+^ response in the lobes occurs sequentially. (Upper panel) GFP fluorescence image of the lobes on one side of the brain (20× objective), followed by the corresponding bioluminescence images. Each panel represents the accumulated light emission occurring during the specified time interval after application of nicotine (time “0”). The first bioluminescence panel shows activation in the α' and β' lobes, which is followed the γ lobe (second panel) and finally, by the α and β lobes (third panel). The outline has been drawn by freehand to highlight the sequential activation of the different lobes. This can also be seen in the associated movies (suppl. movies 5A & B). Resolution is 256×256 pixels, each pixel is approximately 2.6 µm^2^. Light emission (photons/pixel) is coded in pseudocolors.

The antenno-glomerular tract (AGT) is cholinergic [19], and carries the main input for learning and memory processes from the antennal lobes to MB-calyx. In P[GAL4] OK107 flies expressing GA in all the KCs, application of nicotine lead to a rapid and transient increase in Ca^2+^ within the calyx and the cell bodies (Figure 3A–B). However, we made the unexpected observation that nicotine also produced a second [Ca^2+^] rise in the projections of the KCs at the level of the lobes, with a peak response occurring up to 15 min later (Figure 3B–E, movie S4) (mean = 8±1 min, n = 13, 8 flies). In contrast, stimulation with KCl did not produce a similar event. On the other hand, lower nicotine concentrations (10 µM) did not always induce a secondary response. However, repeated applications separated by approximately 15 minutes could yield the delayed secondary response, suggesting that this response could be launched by an accumulative effect (data not shown). A similar secondary response could also be induced by application of the endogenous chemical, acetylcholine (Figure 3E).

### The delayed secondary Ca^2+^ rise occurs sequentially in the MB lobes

Genetic and developmental studies undertaken on the MBs describe differential gene expression and sequential development of the lobes [20]–[22]. Indeed, vertical lobes can be subdivided into two groups of fibers, α and α' lobes (which have their counterparts in medial lobes, as β and β'). From fluorescence images taken of the frontal view, the two subdivisions are visible. In particular, the α' lobe terminates laterally to the α lobe. However, the β and β' lobes are more difficult to discern from one another, and from the γ lobe within the medial lobe (see Figure 1A). The bioluminescent images acquired had a resolution of approximately 1.29 µm^2^ per pixel and they were well superimposed with the fluorescence image. In experiments where the subdivisions could be more clearly identified, the secondary response occured sequentially in the lobes: first in the α'/β' lobes, followed by the γ lobe, and finally in the α/β lobes (Figure 3E & movies S5A & S5B). Indeed, the delayed response observed in the vertical lobe was first seen laterally, which corresponds to the α' lobe, and accordingly in its counterpart in the medial (β') lobe. Thereafter, the response was observed in the medial lobes, corresponding to the γ lobe, since this does not have a vertical counterpart. Finally, the last component of the response occurred in the vertical (α) lobe, and then in the medial (β) lobe (Figure 3F, movies S5).

### Thapsigargin sensitive intra-cellular stores play a role in the delayed secondary Ca^2+^ response

**Figure 4:**
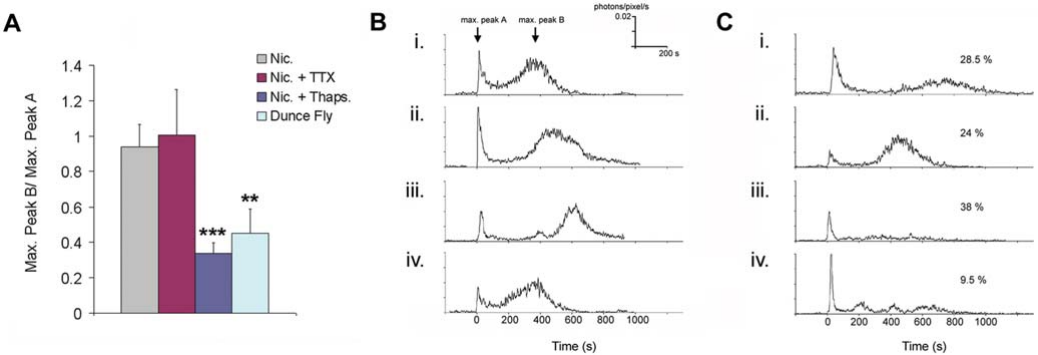
The nicotine induced delayed secondary Ca^2+^ response in the MB lobes is dependent on thapsigargin-sensitive Ca^2+^ stores and is reduced in the *dunce* mutant. A) Histogram showing the means+/−s.e.m. for the calculated ratio of Peak Max. B/Peak Max. A (see methods for explanation), recorded in the medial lobes after application of nicotine (n = 13, 8 flies). The delayed secondary response is significantly blocked by thapsigargin (2 µM, n = 10, 5 flies), but is unaffected by TTX (n = 6, 4 flies). Finally, in *dunce^1^* mutant flies, the delayed secondary response is significantly reduced (n = 16, 11 flies). **P<0.01, *** *P*<0.001. B & C) Four representative individual traces showing the light emission in the medial lobes (photons/pixel/s), from wild-type Canton-S flies (B) and from *dunce^1^* mutant flies (C). The percentage of flies producing responses similar to those represented by each graph is also given.

Homeostatic mechanisms usually prevent prolonged and sustained rises of [Ca^2+^] in physiological conditions. The amplitude of the light intensity corresponding to the secondary response was usually relatively low and total light was therefore plotted using a minimum of 1 second time integrals. The delayed Ca^2+^ response (slow rate of rise and decay) may therefore arise from Ca^2+^-oscillations, which could not be temporally resolved with the sensitivity of this technique. In mammals, intra-cellular Ca^2+^ release from the ER has already been reported to be involved in Ca^2+^-oscillations regulating gene expression and in functions such as synaptic plasticity [23]. In the following studies, it was determined if these Ca^2+^ oscillations were sensitive to the irreversible inhibitor of Ca^2+^ ATPase, thapsigargin [24], which depletes Ca^2+^ levels within the endoplasmic reticulum (ER). Thapsigargin was found to significantly reduce the secondary response caused by nicotine application (Figure 4A, movie S6). In contrast, TTX did not affect the primary or secondary response, ruling out direct activation of the lobes by other putative afferent neurons that send their projections (as pre-synaptic afference) to the MB lobes [25] (Figure 4A). Taken together, these experiments suggest that the delayed Ca^2+^ response involves the participation of the ER compartment within the Kenyon cells of the MBs.

### The delayed secondary Ca^2+^ response after nicotine stimulation is reduced in the *dunce* mutant

In *Drosophila*, genetic approaches have allowed the identification of genes associated with MB physiology, which are implicated in olfactory learning and memory processes [14], [26]. The learning and memory gene *dunce* (*dnc*), encodes for a phosphodiesterase (PDE). This enzyme regulates the level of cAMP [27], which is largely concentrated in the neuropil of the MBs [28]. In further studies, the UAS-GA driven by the OK107 line was expressed in *dnc^1^* mutant flies and the delayed secondary rise in Ca^2+^ induced by nicotine was found to be reduced by a mean of about 50% in the *dnc^1^* mutant fly (Figure 4A, B & C, movie S7).

## Discussion

Described here is a new approach for *in vivo* whole brain imaging of Ca^2+^ signalling in the fly brain using the bioluminescent photoprotein GFP-aequorin. The optical detection of Ca^2+^-induced bioluminescence does not require light excitation and is therefore less invasive than fluorescence imaging. Studies here show that whole brain bioluminescence imaging enables the detection of transient rises in cytosolic Ca^2+^ concentrations occuring in specific neuronal ensembles. In addition, the bioluminescent responses have an excellent signal to noise ratio allowing Ca^2+^ signalling to be monitored in deep brain structures (e.g. the eb, a sub-structure of the CC). This approach can also be applied to pharmacological investigations and studies on mutants.

The use of a photon-counting based technique in bioluminescence imaging eliminated the necessity to pre-select exposure times and recording durations, so that Ca^2+^ signals could be monitored over a wide dynamic temporal range for which they are known to operate. This would incorporate Ca^2+^ signals that may be in control of long term cellular changes, such as gene transcription, synaptic plasticity or cellular proliferation. The spatial resolution of whole brain bioluminescence imaging is dependent on both the light scattering caused by tissue and on the resolution of the camera. Despite the moderate resolution of images acquired in these studies, the origin of the signal is known from genetic targeting and corresponding expression patterns of the reporter. However, the spatial resolution could be improved by using higher magnifications. In an integrated system like the fly brain, bioluminescence imaging could also be useful for defining the spatial and temporal parameters of Ca^2+^ signals linked to known functions. Once these parameters are identified, probably that higher spatial and temporal resolution of the signal could be sort with fluorescence approaches.

In flies, the MBs have been implicated in several functions, such as learning and memory [14], [26], and locomotor activity [29], [30]. In a former study it was reported that all types of MB neurons respond to acetylcholine [5]. However, these studies did not show the secondary response nor the sequential activation of the lobes. This strongly suggests that the secondary Ca^2+^ response in specific neuronal ensembles was identified because of the method employed, which involved continuous recording of Ca^2+^ signals over a long period and selection of time integrals from 50 milliseconds to 10′s of seconds offline.

Ca^2+^ oscillations having a 4 min period have previously been reported in the MBs with transgenic flies expressing aequorin alone [24]. These oscillations were reported to occur spontaneously and were not sensitive to thapsigargin, while they were blocked by TTX. We did not observe such oscillations. However, we worked *in vivo*, while Rosay *et al.,* worked on an *in-vitro* brain preparation.

The secondary Ca^2+^ rise was not related to toxicity, because the delayed secondary response could be observed after successive applications of both nicotine and acetylcholine. In physiological conditions, prolonged and sustained rises in Ca^2+^ are usually toxic for the cell. One explanation is that the observed secondary rise in Ca^2+^ after nicotine stimulation could be made up of fast Ca^2+^-oscillations linked to long-term changes, such as gene expression or synaptic plasticity. IP_3_ signalling has been identified as the driving element for cytosolic Ca^2+ ^oscillations in some model systems. Indeed, we found that the secondary response was significantly reduced by the Ca^2+^-ATPase inhibitor, thapsigargin, which depletes IP_3_ sensitive stores of Ca^2+^.The delayed rise in Ca^2+^ was also found to be reduced in the *dnc* mutant by a value that is in the same range of PDE reduction that is reported for the *dnc^1^* mutant [31]. Extensive genetic and molecular studies indicate that the *dnc* gene is highly complex [32]. Although the de-regulation of cAMP signalling pathways caused by this mutation are poorly characterised, it is known that the *dnc* mutation causes abnormalities in several learning paradigms and alters synaptic transmission and growth cone motility. Repetitive exposures to nicotine have also been shown to induce a hyper-responsiveness that is dependent on cAMP signalling and known to be sensitive to the *dnc* mutation [33]. Thus, the *dnc* sensitive delayed secondary Ca^2+^ response occurring specifically in the MB lobes may play a critical role in cellular processes (synaptic transmission and/or synaptic plasticity) contributing to memory formation.

To date, functional imaging on structures located deep in the *Drosophila* brain, such as the ellipsoid-body of the CC has never been reported with other approaches. Results here show bioluminescence imaging of Ca^2+^ signals using GFP-aequorin can allow the functional characterisation of these deep brain structures. Using a photon counting based technique also allowed images to be analysed with variable time integrals so that the delayed secondary response could be readily identified, despite its relatively low amplitude and prolonged time course. Whole brain bioluminescence imaging can therefore reveal widely dynamic spatial and temporal parameters of the Ca^2+^ signalling system in the living organism. More sensitive techniques (e.g. fluorescence imaging) could then be applied to obtain greater spatiotemporal resolution of the Ca^2+^-signal in structures, such as the mushroom bodies. Anatomo-functional mapping of the *Drosophila* brain using this *in-vivo* genetic approach will therefore provide important information for studies on complex neural pathways, involved in learning and memory, locomotor activity, circadian rhythms and olfaction.

## Materials and Methods

### Flies

All *Drosophila melanogaster* lines were maintained at 24°C on standard food medium. P[GAL4] OK107 line and *dunce^1^* mutant was obtained from the Bloomington *Drosophila* Stock Centre, C232 by D. Armstrong and K. Kaiser. All flies were backcrossed six fold with Canton-S to standardize the genetic background (Cantonization).

### GFP-aequorin construct and genesis of transgenic flies

The GFP-aequorin (GA) insert of pG5A [9], was cut out with EcoRI and XhoI and inserted into the pUAST vector [13]. Germ-line transformations of Canton-S white (-) flies (WCS10) were generated using standard techniques. The transformants were crossed with a MB-specific GAL4 driver line, OK107 [7], to examine the expression pattern. Three transformation lines were shown to have similar fluorescence. The three lines have been tested for their bioluminescence and as they were found to give similar results, the line UAS-GA-2 was chosen for studies reported here.

### Preparation of flies for recording

Brains were exposed by partial removal of the head capsule. During this procedure, the underlying neural sheath is also removed in order to expose the outer surfaces of the brain. The flies are mounted upside down in an acrylic block, which is then mounted inside a diamond shaped slice chamber (Harvard Apparatus). The slice chamber containing the prepared fly was then transferred to an inverted microscope for recording. Recordings were undertaken in a fly ringers buffer containing, 130 mM NaCl, 5 mM KCl, 2 mM MgCl_2_, 2 mM CaCl_2_, 36 mM Sucrose, 5 mM Hepes-NaOH, pH 7.3 [3]. The exposed fly brains are first incubated for more than 1 hour at room temperature in the fly ringers containing 5 µM native coelenterazine (Interchim, France). Following incubation, the chamber containing the mounted fly is washed to remove excess coelenterazine and the fly ringers with or without added drugs was then delivered to the chamber by perfusion (1 ml/min by gravity flow). A schematic drawing of the set-up is provided, see Figure S1. The fly is able to breathe via the tracheal system and can be maintained for more than 12 hours in this condition.

### Detection of Ca^2+^-induced bioluminescence activities

Detection of Ca^2+^-induced bioluminescence activity has been previously described [10]. Briefly, a highly sensitive image photon detector (IPD 3, Photek Ltd. East Sussex, UK), which produces very low background counts (<1 photon/s in a 256×256 pixel region), was utilized for detection of bioluminescence (50 ms time window). Observations were made using a 20× objective with a high numerical aperture (0.5, Carl Zeiss, Germany). Resolution of the system was 256×256, with each pixel being equal to 1,2875 microns^2^.

### Preparation of drugs

Nicotine was prepared as a stock solution (100 mM) and diluted further in fly ringers (see composition above) to a concentration of 100 µM. KCl was prepared in fly ringers to a concentration of 70 mM and the concentration of NaCl was adjusted accordingly. Thapsigargin was dissolved in 100% DMSO and diluted to a final concentration of 2 µM (final DMSO concentration 0.2%). TTX was stored as a 1 mM stock solution and diluted to 1 µM in fly ringers. Verapamil was dissolved in fly ringers to a concentration of 10 µM. All drugs were purchased from Sigma.

### Statistical analyses

Data was analyzed in Excel and statistical significance was determined using a one-tailed student t test, with unequal variance.

## Supporting Information

Figure S1 Schematic drawing of the setup. Setup used to monitor and quantify the bioluminescence light emitted by the Ca2+activated GFP-aequorin. An inverted microscope is equipped with a CCD camera, allowing a fluorescent image of GFP expression in the whole brain to be recorded. Then, by switching off an automatised mirror, this allows the light emitted to directly reach the photon counting camera. Each photon emitted are detected and the X,Y coordinates as well as its time (t) are recorded. After the recording session, the data are analysed by performing various desired and appropriated integration time to visualize the neuronal activity. Remarks that because the photon detector (IPD) is placed below the preparation (inverted microscope), this limits our approach to pharmacological manipulations (similar to a perfusion bath) or to spontaneous recording events. An upright built system will allow physiological and even behavioural manipulations, like odor presentation, locomotor activity recording or else (works in progress).
(1.02 MB TIF)

Movie S1 *In-vivo* bioluminescence imaging of K^+^-depolarised Ca^2+^ uptake in the MBs. Ca^2+^ uptake in the mushroom bodies (MBs) was directly visualised after K^+^-depolarisation of the fly brain. Each frame represents 60 s of light accumulation and is shifted by 10 s (6 frames/s). The movie is seen 60 times faster. The light emission is coded in pseudocolors (0-5 photons/pixel) (QuickTime; 483 Ko).
(0.49 MB MOV)

Movie S2 *In-vivo* bioluminescence imaging of K^+^-depolarised Ca^2+^ uptake in the ellipsoid-body. Ca^2+^ uptake in the ellipsoid-body and cell bodies (a substructure of the central complex) was directly visualised after K^+^-depolarisation of the fly brain (70 mM KCl). Each frame represents 60 s of light accumulation and is shifted by 10 s (6 frames/s). The movie is seen 60 times faster. The light emission is coded in pseudocolors (0-5 photons/pixel) (QuickTime; 663 Ko).
(0.68 MB MOV)

Movie S3 Spontaneous activity in the ellipsoid-body, a sub-structure of the central complex. Each frame represents 120 s of light accumulation and is shifted by 30 s (6 frames/s). The movie is seen 180 times faster. The light emission is coded in pseudocolors (0-5 photons/pixel) (QuickTime; 789 Ko).
(0.81 MB MOV)

Movie S4 Ca^2+^-signalling in the MBs after application of nicotine. Nicotine (100 µM) induces transient increase in [Ca^2+^]_i_ in all parts of the MBs (OK107) and a delayed secondary Ca^2+^ response in the lobes 4 to 15 min after the primary response. Each frame represents 60 s of light accumulation and is shifted by 10 s (6 frames/s). The movie is seen 60 times faster. The light emission is coded in pseudocolors (0-5 photons/pixel) (QuickTime; 992 Ko).
(1.02 MB MOV)

Movie S5A Sequential activation of the delayed Ca^2+^-response in the MB lobes. The nicotine induced delayed secondary response in the MB lobes occurs sequentially, first, in the α'/β' lobes, second, in γ lobes, and finally in α/β lobes. Each frame represents 60 s of light accumulation and is shifted by 10 s (12 frames/s). The movie is seen 120 times faster. The light emission (photons/pixel) is coded in pseudocolors (2-10 photons/pixel) (QuickTime; 1328 Ko).
(1.36 MB MOV)

Movie S5B Sequential activation of the delayed Ca^2+^-response in the MB lobes. The nicotine induced delayed secondary response in the MB lobes occurs sequentially, first, in the α'/β' lobes, second, in γ lobes, and finally in α/β lobes. Each frame represents 90 s of light accumulation and is shifted by 5 s (12 frames/s). Resolution is 256×256. The light emission (photons/pixel) is coded in pseudocolors (1-6 photons/pixel) (QuickTime; 2718 Ko).
(2.78 MB MOV)

Movie S6 Thapsigargin blocks the nicotine induced delayed Ca^2+^-response in the MB lobes. Application of thapsigargin, 15 min before and during the nicotine application (100 µM) blocks the secondary response in the MB lobes, suggesting that this delayed response requires the intra-cellular calcium store in the endoplasmic reticulum. Each frame represents 60 s of light accumulation and is shifted by 10 s (6 frames/s). The movie is seen 60 times faster. The light emission (photons/pixel) is coded in pseudocolors (0-5 photons/pixel) (QuickTime; 536 Ko).
(0.55 MB MOV)

Movie S7 The nicotine induced delayed Ca^2+^-response in MB lobes is reduced in *dunce* mutant flies. The nicotine (100 µM, 1 min) induced delayed secondary response in MB lobes is reduced and in some cases, absent in *dunce^1^* mutant. As a positive control, KCl was applied (70 mM, 1 min) 30 min after nicotine application to verify that the Kenyon cells of the MBs are still physiologically functional. This induced a strong response simultaneously in all parts of the MBs, including the complex calyx/cell-bodies as well as in the medial and vertical lobes. Each frame represents 60 s of light accumulation and is shifted by 10 s (6 frames/s). The movie is seen 60 times faster. The light emission (photons/pixel) is coded in pseudocolors (1-8 photons/pixel) (QuickTime; 838 Ko).
(0.86 MB MOV)
