# Supplementary figures and images for: In vivo Bioluminescence Imaging of Ca2+ Signalling in the Brain of Drosophila
Source: PLoS One. 2007 Mar 7;2(3):e275. doi: 10.1371/journal.pone.0000275 (PMC1803028; doi:10.1371/journal.pone.0000275)

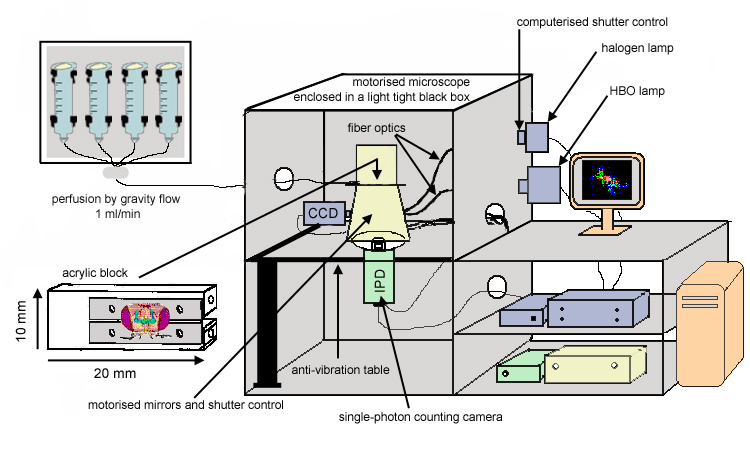

Supplement: Figure S1 — Schematic drawing of the setup. Setup used to monitor and quantify the bioluminescence light emitted by the Ca2+activated GFP-aequorin. An inverted microscope is equipped with a CCD camera, allowing a fluorescent image of GFP expression in the whole brain to be recorded. Then, by switching off an automatised mirror, this allows the light emitted to directly reach the photon counting camera. Each photon emitted are detected and the X,Y coordinates as well as its time (t) are recorded. After the recording session, the data are analysed by performing various desired and appropriated integration time to visualize the neuronal activity. Remarks that because the photon detector (IPD) is placed below the preparation (inverted microscope), this limits our approach to pharmacological manipulations (similar to a perfusion bath) or to spontaneous recording events. An upright built system will allow physiological and even behavioural manipulations, like odor presentation, locomotor activity recording or else (works in progress). (1.02 MB TIF) [file pone.0000275.s001.tif]
